# IMPROVED EXPERIMENTAL MODEL TO EVALUATE SUBMUCOSAL INJECTION SOLUTIONS FOR ENDOSCOPIC SUBMUCOSAL DISSECTION

**DOI:** 10.1590/S0102-6720201500040011

**Published:** 2015

**Authors:** Kendi YAMAZAKI, Fauze MALUF-FILHO, Vitor Alves Pessoa da COSTA, Fernanda Cristina Simões PESSORRUSSO, Fabio Yuji HONDO, Paulo SAKAI, Luis Francisco Poli de FIGUEIREDO

**Affiliations:** 1 Department of Gastroenterology - Gastrointestinal Endoscopy Unit, São Paulo University Medical School; 2 Experimental Laboratory 26 - São Paulo University Medical School, São Paulo, SP, Brazil

**Keywords:** Early gastric cancer, Endoscopic mucosal resection, Endoscopic submucosal dissection

## Abstract

***Background*:**

Endoscopic submucosal dissection carries an increased risk of bleeding and perforation. The creation of a long lasting submucosal cushion is essential for the safe and complete removal of the lesion. There is not a suitable experimental model for evaluation of the durability of the cushioning effect of different solutions.

***Aim*:**

To describe an improved experimental model to evaluate submucosal injection solutions.

***Methods*:**

A total of four domestic pigs were employed to evaluate two different submucosal fluid solutions in the gastric submucosa. After midline laparotomy, the anterior gastric wall was incised from the gastric body to the antrum and its mucosal surface was exposed by flipping inside out the incised gastric wall. Two different solutions (10% mannitol and normal saline) were injected in the submucosa of the anterior wall of the distal gastric body. All submucosal cushions were injected until they reach the same size, standardized as 1.0 cm in height and 2.0 cm in diameter. A caliper and a ruler were employed to guarantee accuracy of the measurements.

***Results*:**

All four animal experiments were completed. All submucosal cushions had the exact same size measured with caliper and a ruler. By using the mannitol solution, the mean duration of the submucosal cushion was longer than the saline solution: 20 and 22 min (mean, 21 min) vs 5 and 6 min (mean, 5.5 min)

***Conclusions*:**

This experimental model is simple and evaluate the duration, size, and effect of the submucosal cushion, making it more reliable than other models that employ resected porcine stomachs or endoscopic images in live porcine models.

## Abstract (pt)

***Racional*:**

Sabe-se que os maiores riscos na ressecção endoscópica da submucosa são o sangramento e a perfuração. A criação de um coxim submucoso duradouro é essencial para a remoção da lesão completa e com segurança. Atualmente não se conhece um modelo experimental adequado para avaliação da durabilidade do coxim submucoso com diferentes soluções.

**Objetivo:**

: Descrever um modelo experimental melhorado para avaliar soluções de injeção de submucosa.

***Métodos*:**

Foram utilizados quatro porcos domésticos para avaliar dois tipos diferentes de soluções para injeção na submucosa gástrica. Após laparotomia mediana, a parede gástrica anterior foi aberta no sentido corpo-antro e sua superfície mucosa foi exposta por eversão da abertura gástrica. Dois tipos diferentes de solução (manitol a 10% e solução salina normal) foram injetados na submucosa da parede gástrica anterior de corpo distal. Todos os coxins submucosos foram injetados até que alcançassem o mesmo tamanho, padronizado como 1,0 cm de altura por 2,0 cm de diâmetro. Foram aplicados régua e compasso para garantir a acurácia das medidas.

***Resultados*:**

O experimento foi completo nos quatro animais. Todos os coxins submucosos tinham o mesmo tamanho, medido com régua e compasso. Com o uso da solução de manitol, a duração média do coxim submucoso foi maior que a da solução salina: 20 e 22 min (média 21 min) vs 5 e 6 min (média 5,5 min).

***Conclusões*:**

Este modelo experimental é simples e permite analisar duração, tamanho e efeito do coxim submucoso, tornando-se mais confiável que outros modelos que empregam estômagos ressecados de porcos ou imagens endoscópicas de modelos porcinos vivos.

## INTRODUCTION

Endoscopic resection techniques such as endoscopic mucosal resection and endoscopic submucosal dissection for the treatment of early gastric cancer are widely practiced in Japan and are gaining acceptance in many other countries^6^^,^ 8. The introduction of these endoscopic modalities lead to improvements in patient quality of life without compromising survival rates. However, endoscopic submucosal dissection carries an increased risk of bleeding and perforation^10^^,^ 9. The creation of a long lasting submucosal cushion is of utmost importance for the safe and complete removal of the lesion. In clinical practice many solutions are used for this purpose. Of the currently available agents, hyaluronic acid, glycerol, and hydroxypropylmethylcellulose appear to have a durable cushioning effect^12^^,^ 3 ^,^ 13 ^,^ 2. 

Experimental studies on animal models describe the benefits of each solution^14^. Most of these experiments are ex vivo studies performed with porcine stomach. In our opinion, this is not a suitable model for evaluation of the durability of the cushioning effect, since vascular flow is compromised in ex vivo models. On the other hand, endoscopic evaluation of the submucosal cushioning effect in live porcine models has the bias of measures obtained by endoscopic bidimensional and magnified images^2^^,^ 4 ^,^ 1.

The aim of this study is to describe an improved experimental model to evaluate submucosal injection solutions on live porcine.

## METHODS

The University of São Paulo University Medical School Ethical Committee approved this study. Animals were kept fasting for approximately 12 hours prior to the procedure and underwent general anesthesia with endotracheal intubation. A total of four domestic pigs (50 kg) were employed to evaluate two different submucosal fluid solutions in the gastric submucosa. Skin preparation was initially obtained with antiseptic detergent followed by the application of an antiseptic solution, chlorexidine gluconate 4%.

**FIGURE 1:**
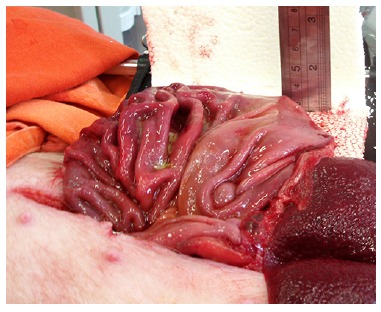
- View of the opened stomach and the vertical ruler used to measure the elevation of the cushion

A midline abdominal incision was made to approach the abdominal cavity and stomach. The anterior gastric wall was incised from the gastric body to the antrum and its mucosal surface was exposed by averting or flipping inside out the incised gastric wall (Figure 1). During this procedure the main gastric vessels, such as the left and right gastric arteries and left and right gastroepiploic arteries, were not compromised.

**FIGURE 2:**
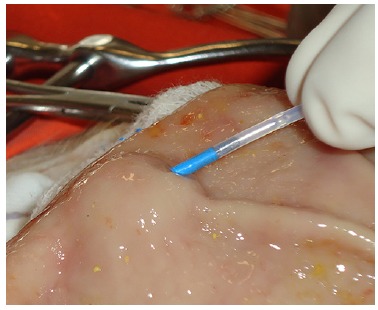
**-** Creation of the submucosal cushion

**FIGURE 3:**
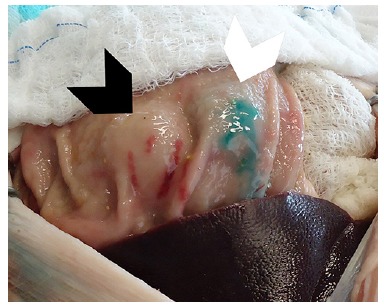
**-** View of the two submucosal cushions created by the injection of 10% mannitol solution (left cushion) and saline solution (right cushion). Mucosal extravasation of the saline solution explains the bluish aspect of the cushion displayed on the right.

**FIGURE 4:**
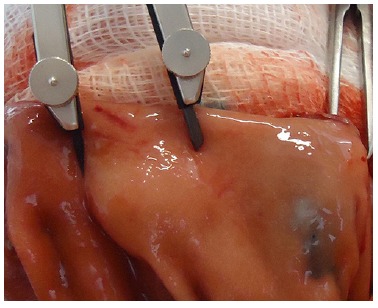
- Measurement of the cushion diameter by the caliper

In all four animals, two different solutions were injected in the submucosa of the anterior wall of the distal gastric body. The first, was as mixture of 100 ml of 20% mannitol, 100 ml of normal saline, and 2.0 ml of 4% indigo carmine dye. The second, was done in a separate site, 5 cm distal from the first injection, using a mixture of 100 ml of normal saline with 2.0 ml of 4% indigo carmine dye. The technique of injection was the same for both solutions. Using a 10 ml syringe filled with the solution and attached to a 23 gauge sclerotherapy needle (Boston Scientific, São Paulo, Brazil), 2.0 ml of the solution was injected through the mucosa into the submucosa (Figure 2). If the mucosa did not lift after a 0.5 ml injection, the needle was repeatedly reinserted at different angles until a visible mucosal elevation was created. All submucosal cushions were injected until they reach the same size, standardized in this study as 1.0 cm in height and 2.0 cm in diameter (Figure 3). A caliper and a ruler were employed to guarantee accuracy of the measurements (Figure 4). The time for cushion disappearance was recorded in a standardized manner by using a stopwatch that was started immediately upon completion of the submucosal injection. The results were expressed in minutes.

After the complete disappearance of the cushion, the incised area of the stomach was sutured and the abdominal wall was closed with separate stitches of 2-0 poliglactin. All animals were kept alive for 6 h and euthanized.

It was not calculated the sample size because the main objective was to describe the improved experimental model and present the time required for the flattening of the cushion created by both solutions in the four animals and expressed those figures in minutes.

## RESULTS

**FIGURE 5:**
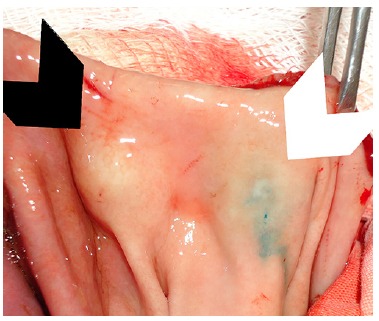
- The left cushion (mannitol) is still protruded while the saline cushion on the right is almost flat

All four animal experiments were completed. All submucosal cushions had the exact same size measured with caliper and a ruler. By using the mannitol solution, the mean duration of the submucosal cushion was longer than the saline solution: 20 and 22 min (mean, 21 min) vs 5 and 6 min (mean, 5.5 min) (Figure 5). The results are detailed at Table 1.

**TABLE:** 1 - Time elapsed until flattening of the submucosal cushion

	**Animal 1**	**Animal 2**	**Animal 3**	**Animal 4**				
	**Mannitol**	**Saline**	**Mannitol**	**Saline**	**Mannitol**	**Saline**	**Mannitol**	**Saline**
Duration (min)	22	5	22	6	20	6	20	5

## DISCUSSION

Several experimental models are used in the evaluation of the duration of the cushioning effect of different solutions injected into the submucosal layer of the gastric wall. High viscoelasticity appears to be an important property of an effective submucosal fluid cushion. Viscoelasticity combines the qualities of viscosity (resisting shear flow when stress is applied) and elasticity (straining when stretched and returning to the original state once the stress is removed).^5^

Using six different solutions as cushioning agents in live pigs, Giday et al. compared the performance and duration of the mucosal elevation by endoscopy, showing different results with each type of agent^4^. Employing the same model, Conio et al. compared normal saline solution, normal saline plus epinephrine solution, 50% dextrose, 10% glycerine/5% fructose and 1% hyaluronic acid for the creation of a submucosal bleb in the distal esophagus^1^. Both studies relied on the endoscopic view of the flattened bleb to determine the duration of the cushioning effect of the injected solution. It is believed that a bidimensional image created by the endoscope may be imperfect if one is trying to evaluate the duration of the submucosal cushion, because size assessment by endoscopic image lacks accuracy and cushions of the same size are desirable for this kind of comparison. Other studies used sacrificed porcine stomachs to evaluate the same objective^7^. The methodology used in these studies consists of injecting the same amount of solution into the submucosal layer, measuring the size of the cushion (in ex vivo models), and recording the time of its disappearance. Using fresh resected human colon specimens, Sumiyoshi et al. compared glycerol and normal saline solution, where submucosal elevations were observed from the lateral position and recorded using a measuring device 1, 3, 5, 7, and 10 min after injection^11^. The glycerol group maintained a significantly longer lasting submucosal elevation. Recently, Yoshida et al. used a similar method but with resected porcine colon and esophagus cut into 10 cm diameter segments to examine the duration of the submucosal cushion using hyaluronic acid and normal saline^15^.

The above-mentioned models share the same limitation of all ex vivo models: there is no vascularization in the tissue, which definitively compromises the hydrostatic and osmotic pressures governing the distribution of solution through the tissue. In a study comparing different solutions, Polymeros et al. used ex vivo porcine models within the first hours of animals' death in order to avoid significant tissue changes; however, this effort does not address the issue of vascularization^7^. 

The model proposed here addresses most of the limitations of other experimental models. However, in the clinical situation of endoscopic submucosal resection, the stomach is fully distended by air and it is arguable whether the intragastric pressure could exert significant pressure and cause any effect on the size or the duration of the submucosal cushion. This could be a significant limitation of this model.

Regardless of which submucosal solution was employed, the main objective of this study was to prove the feasibility of the proposed model. Indeed the expected differences obtained with the different solutions attest to the reliability of the model. 

Is recognized that these results do not differ significantly from other studies results. This fact could argue against the need for an improved model to evaluate submucosal injection solutions for endoscopic submucosal dissection. On the other hand this model is robust as it addresses most of the inherent limitations of other models: direct view and measurement of the submucosal cushion, preservation of the vascular structures. It might prove critical for the evaluation of future solutions.

## CONCLUSION

This experimental model to evaluate submucosal injection solutions in live porcine models is simple and enabled to evaluate the duration, size, and effect of the submucosal cushion, making it more reliable than other models that employ resected porcine stomachs or endoscopic images in live porcine models.
